# It’s complicated: characterizing the time-varying relationship between cell phone mobility and COVID-19 spread in the US

**DOI:** 10.1038/s41746-021-00523-3

**Published:** 2021-10-27

**Authors:** Sean Jewell, Joseph Futoma, Lauren Hannah, Andrew C. Miller, Nicholas J. Foti, Emily B. Fox

**Affiliations:** Apple, One Apple Park Way, Cupertino, CA 95014 USA

**Keywords:** Respiratory tract diseases, Infectious diseases, Statistics, Epidemiology

## Abstract

Restricting in-person interactions is an important technique for limiting the spread of severe acute respiratory syndrome coronavirus-2 (SARS-CoV-2). Although early research found strong associations between cell phone mobility and infection spread during the initial outbreaks in the United States, it is unclear whether this relationship persists across locations and time. We propose an interpretable statistical model to identify spatiotemporal variation in the association between mobility and infection rates. Using 1 year of US county-level data, we found that sharp drops in mobility often coincided with declining infection rates in the most populous counties in spring 2020. However, the association varied considerably in other locations and across time. Our findings are sensitive to model flexibility, as more restrictive models average over local effects and mask much of the spatiotemporal variation. We conclude that mobility does not appear to be a reliable leading indicator of infection rates, which may have important policy implications.

## Introduction

In the hopes of better informing public health decision-making, researchers have developed many prediction models to forecast the COVID-19 pandemic. Effective forecasts capable of identifying reliable leading indicators of emerging outbreaks could improve policy recommendations. To this end, factors such as mask-wearing^1,2^, weather^3,4^, and demography^5^ have been found to be associated with rates of infection in the United States. The effectiveness of other non-pharmaceutical interventions (NPIs) such as government lockdowns is also well studied^6–8^, although some questions still remain. For instance, it is challenging to disentangle the effects of overlapping NPIs, such as the rapid increase in mask-wearing in early April 2020 alongside widespread lockdowns in many parts of the United States.

Cell phone mobility data has emerged as an appealing surrogate of government mandates. Since it is a directly observable measure of human movement, it contains more information than the duration of government orders. In addition, it may serve as a better proxy for the actual quantity that government actions are intended to reduce: the relative frequency of risky in-person interactions where transmissions may occur. Mobility information is available through public APIs such as Google’s Community Mobility Reports^9^ and SafeGraph’s completely at-home metric^10^. The ubiquity of accessible mobility data, and the lack of alternative sources of data—such as contact tracing information—has made mobility an attractive proxy for interactions.

As mobility plummeted to unprecedented levels during the first wave of the pandemic, these publicly available data sources received widespread attention. Mainstream media such as the Washington Post^11,12^, Wall Street Journal^13^, New York Times^14^, Los Angeles Times^15^, and National Public Radio^16^ have all analyzed cell phone mobility and highlighted its record drop in 2020. Moreover, public-facing epidemiology dashboards, such as the US CDC and Prevention^17^ and the Institute for Health Metrics and Evaluation^18^, prominently list mobility as a metric of interest. As articles in leading scientific journals began to suggest that mobility data could be a valuable tool for battling the pandemic^19–21^, it is not surprising that many COVID-19 forecasts have used mobility as a data source.

Although there is a large body of work using mobility to predict COVID-19 spread, many of their conclusions are not broadly applicable outside of the initial wave of the pandemic. In particular, data limitations and inherent modeling assumptions restrict the applicability of these earlier works^8,21–25^. As the pandemic evolved, an obvious limitation is that early papers only looked at data from the first few months through June 2020^8,21–24^. Furthermore, most articles limited the set of locations modeled to a small number of major cities^21,22^, or fit models at a coarser state level^8,25^. Such limitations in the length of time and number of locations modeled render these works incapable of making inferences about local outbreaks across time. Another key limitation in most prior work—with one exception^24^—is the overly restrictive assumption that the relationship between mobility and infection rates is stationary. Although this stationarity assumption was reasonable during the initial wave of the pandemic, large shifts in behavior due to evolving government guidance and adherence to such guidance suggest that coarse mobility may no longer be a good proxy for potentially risky transmission events^26,27^; as such, the relationship between mobility and infection rates today likely differs from spring 2020.

Capturing the time-varying relationship between mobility and infection rates is especially challenging due to the incomplete, heterogeneous, and non-stationary nature of the data. For instance, the lack of reliable data on adherence to mask-wearing during the beginning of the pandemic in spring 2020 makes it difficult to identify the relationship between mask-wearing and infection rates. This problem is exacerbated by the fact that it is important to adjust for mask-wearing when interpreting the effect of mobility on growth rates. Reported case data come with their own set of unique challenges, including highly variable reporting delays, strong day-of-week effects, and differential rates of testing. Moreover, since we only observe this data over a relatively short time frame, it is difficult to adjust for seasonality.

To assess the temporal and spatial utility of mobility data in this challenging data setting, a central objective of this work is to identify a flexible yet interpretable class of models that can sufficiently disentangle how the effect of mobility changes over time and space. To this end, we show that restrictive models effectively average over local effects by naively assuming a constant relationship between mobility and transmission. Conversely, we show that overly flexible models lead to spurious correlations and conclusions.

Our proposed multilevel regression model strikes a balance: we allow the association between mobility and growth rates to vary across groups of nearby counties and over four distinct “waves” of 13 weeks each. The granularity of this spatial clustering and temporal variation of coefficients is critical to the robustness of our inferences. We analyze an entire year of data across 94% of all 3143 US counties (covering 99.7% of the total population) and use Google’s Mobility Trends as our measure of mobility. We replicate prior work that found strong first wave associations between mobility and infection rates. Furthermore, we find that the strength of this association is strongest in the most populous counties, but is otherwise highly variable across geographies, and significantly weakens after the first wave.

## Results

### Visualization of mobility measures and infection growth rates over time

**Fig. 1:**
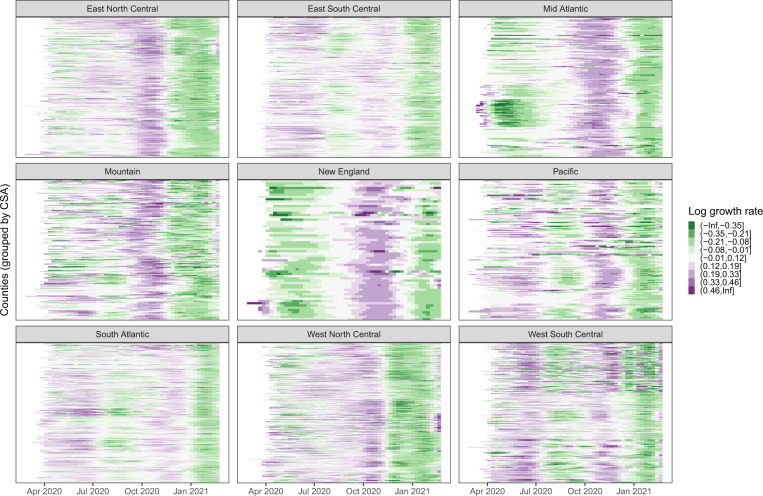
Weekly log infection growth rate *y*_*i*,*t*_ of the inferred true incidence of infection. Each county *i* is faceted into panels by its US Census division. Within a panel, each row represents a county, and counties in the same combined statistical area (CSA) are grouped together in adjacent rows. For example, the cluster of rows in the bottom third of the Mid Atlantic with rapidly declining growth rates in April 2020 represent counties in the New York-Newark CSA. Counties within the same CSA tend to exhibit similar trends in log growth rate. At a high level, the national surge in fall 2020, followed by declining infection rates in early 2021 is pronounced.

We first examine weekly county-level mobility and infection growth rate trends. Figure 1 visualizes the estimated weekly infection growth rate of the inferred true incidence of infection for each of the 2951 US counties modeled. Counties are displayed according to the nine US Census divisions: New England, Mid Atlantic, East North Central, West North Central, South Atlantic, East South Central, West South Central, Mountain, and Pacific; a map of Census Regions and Divisions is available from the US Census^28^. Within a division, counties falling in the same combined statistical area (CSA—a grouping of counties connected by workplaces and commuting patterns) appear in adjacent rows. Counties in the same CSA tend to exhibit similar growth rates, as evidenced by the clear clustering patterns in growth rates. Different waves of the pandemic across divisions are also apparent.

**Fig. 2:**
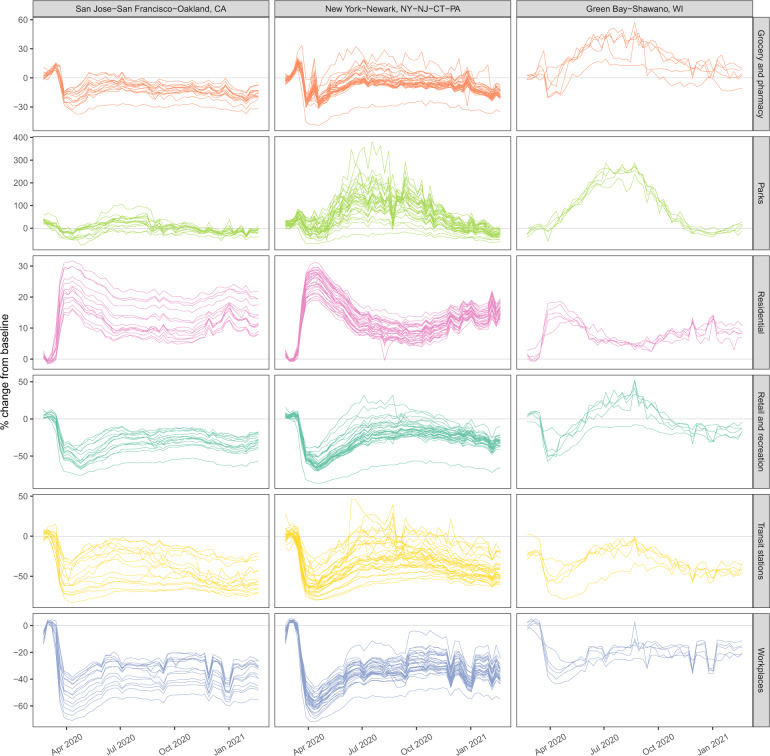
Illustrative county-level mobility. County-level weekly % change from baseline mobility for six mobility categories (grocery and pharmacy, parks, residential, retail and recreation, transit stations, and workplace) are shown for three CSAs.

Google’s mobility trends capture six distinct types of mobility: grocery/pharmacy, residential, retail/recreation, workplace, transit, and parks. Figure 2 shows the weekly trend for each of these variables for each county in three CSAs: New York City, San Francisco, and Green Bay, WI. Mobility values are reported relative to a baseline level in January 2020 for each county, which normalizes for population and pre-pandemic mobility levels. The rapid drop in mobility following widespread lockdowns in March 2020 is present in all locations. Furthermore, it is clear that these six mobility variables are tightly connected: grocery/pharmacy, retail/recreation, workplace, and transit are positively correlated, while residential mobility is negatively correlated with the others.

### Overly flexible models lead to incorrect and misleading inferences

**Fig. 3:**
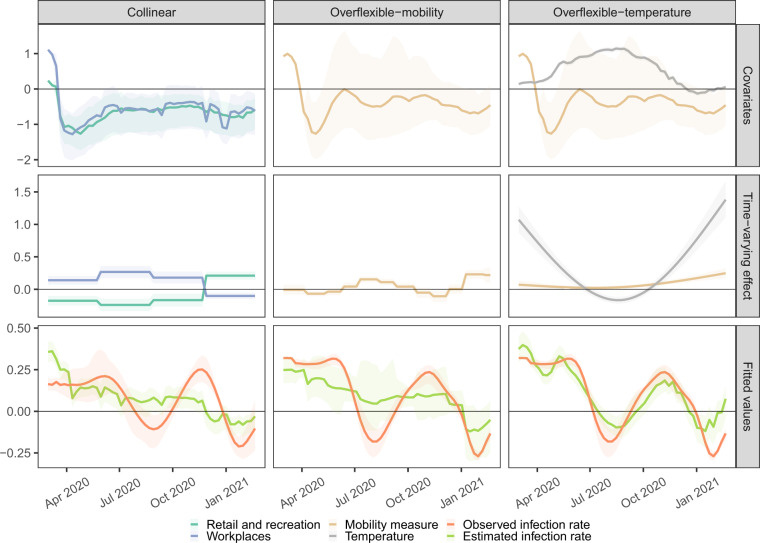
Illustrative model shortcomings. Pitfalls due to collinearity in covariates (left), too much flexibility in mobility (middle), and too much flexibility by including temperature (right). Observed covariates and infection rates from two counties are used to demonstrate these limitations; the same data is used for both overflexibile examples. Observed covariate values (top), estimated time-varying effects (middle), and fitted and observed growth rates (bottom) are shown with median (solid lines) and 95% quantiles (shaded).

Before presenting results from our final model, we begin with some examples of how overly flexible models can overfit and lead to confusing conclusions. Each column of Fig. 3 highlights a different type of pitfall that can occur when the models under consideration are not properly constrained. The top row of the figure plots specific predictor variables (e.g., temperature or mobility) of interest. The middle row shows the estimated coefficients learned by a model for the variables in the top row. The bottom row shows the observed and fitted (i.e., predicted) values from the model.

The left column (labeled “collinear”) of Fig. 3 illustrates an issue caused by collinear input predictor variables. It is tempting to include each of the Google mobility types as separate variables in a model that predicts weekly infection growth rates from covariates such as mobility. However, the strong correlations between the different mobility variables often lead to misleading estimated associations between distinct mobility variables and infection growth rates. The top-left pane of the figure displays the retail/recreation and workplace mobility, two highly correlated mobility metrics within this CSA. Nonsensically, the learned association between retail mobility and infection rates is negative throughout the first three waves. This misleadingly suggests that higher levels of retail-related mobility correlate with lower infection rates, but is clearly an artifact of the collinearity between retail and workplace mobility. In our final model, we collapse the original six Google mobility measures into a single value using principal components analysis to avoid such unintentional side effects caused by collinearity^29^. This univariate feature captures over 60% of the variability in the original six mobility measures.

The middle (“overflexible-mobility”) column of Fig. 3 identifies another pitfall caused by too much model flexibility. The first principal component of mobility is plotted, along with its estimated association with growth rates from a model that allows for the association to varying freely each month. The model’s effect of mobility over time for this location varies considerably and appears to be overfit.

The right (“overflexible-temperature”) column Fig. 3 shows results from a different model, now allowing both the effect of temperature and the univariate measure of mobility to vary smoothly over time. Allowing the effect of temperature to also vary over time overpowers much of the signal contained in mobility, and it is clear that the model is overfitted by the near-perfect fit observed.

### Properly constrained models lead to meaningful inferences

We incorporated the findings from these pitfalls into our final model. We use the first principal component of mobility as a univariate summary of the original six mobility metrics, and allow its effect to vary over four “waves” of 13 weeks each, spanning February 2020 to February 2021. Full details of the model can be found in Methods.

**Fig. 4:**
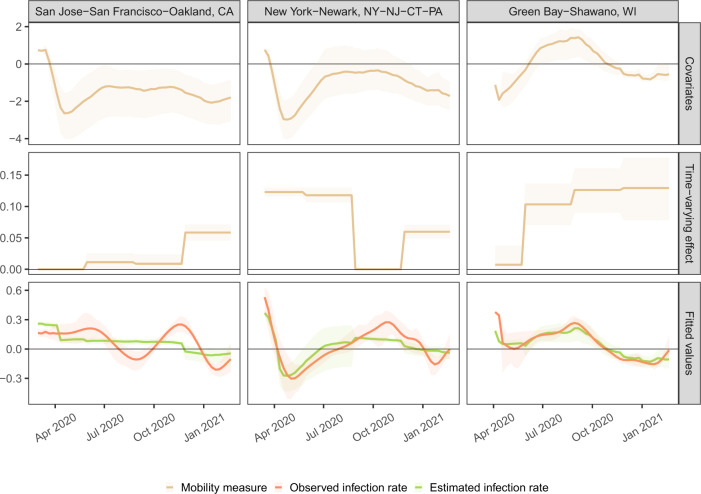
Illustrative model data, estimated effects, and fitted values. Mobility covariates (top), estimated time-varying effect of mobility (middle), and fitted and observed infection growth rate values (bottom) for three examples CSAs. New York has a strong estimated effect of mobility in waves one and two, whereas Green Bay has a strong estimated effect of mobility in the second to fourth waves. San Jose has a moderate effect of mobility in the fourth wave. Median (solid lines) and 95% quantiles (shaded) are shown.

As the first set of qualitative checks, we display in Fig. 4 how well our final model fits the observed infection rates for three CSAs chosen to illustrate heterogeneity in conclusions and model fit. Each column shows results from San Francisco, New York City, and Green Bay, WI. For each location, the aggregate mobility metric is plotted over time, along with the model’s coefficients for mobility per wave and the fitted and observed infection rate values. New York had a strong association between mobility and growth rates at the beginning of the pandemic, Green Bay had a strong association later in the pandemic, and San Francisco never had a strong association.

### Mobility was most predictive in urban areas during spring 2020; elsewhere exhibited substantial variation

**Fig. 5:**
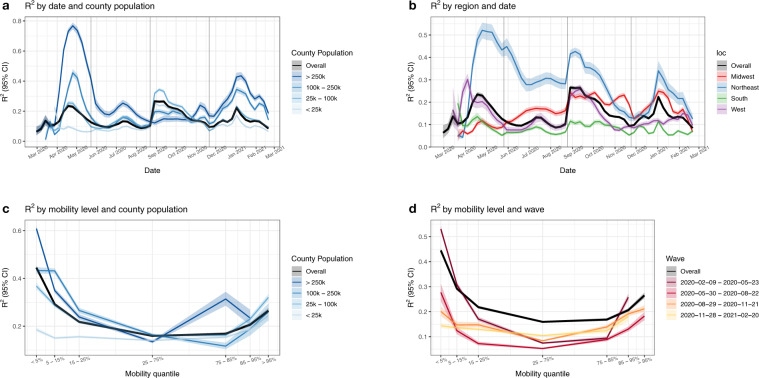
Model performance. **a** *R*^2^ per week, overall, and by county population. **b** *R*^2^ per region and overall. At these coarse levels, models fit the best during April–May 2020. Fits were poor in summer and improved in some places during fall and winter, but never return to the initial high levels. **c** *R*^2^ as a function of the overall level of mobility, further broken down by county population. Mobility contains the most signal in the highest population counties when its overall value is extremely low. **d** *R*^2^ as a function of the overall level of mobility, further broken down by a wave. Mobility contains the most signal in the first wave at extremely low values. Median (solid lines) and 95% quantiles (shaded) are shown.

Figure 5 presents the *R*^2^ of our model across different subsets of data. Panel (a) shows the overall *R*^2^ of the model for each week and the *R*^2^ across counties with varying population sizes. The overall fit is best during the first months of the pandemic and for the largest counties (populations of more than 250,000, comprising 64% of the total US population). *R*^2^ is low across the rural 46% of counties with a population of less than 25,000. Panel (b) shows similar *R*^2^ results according to the US Census region. The Northeast exhibits the best fit while the South has the poorest fit. Panels (c) and (d) show additional *R*^2^ results as a function of overall relative mobility levels across all locations and time. Model performance is highest during the first wave in most urban counties when mobility levels are at their lowest values. Interestingly, during the third and fourth waves, there is minimal difference in *R*^2^ as a function of mobility levels, suggesting that at this coarse level of analysis mobility’s association with infection growth rates weakened over time.

**Fig. 6:**
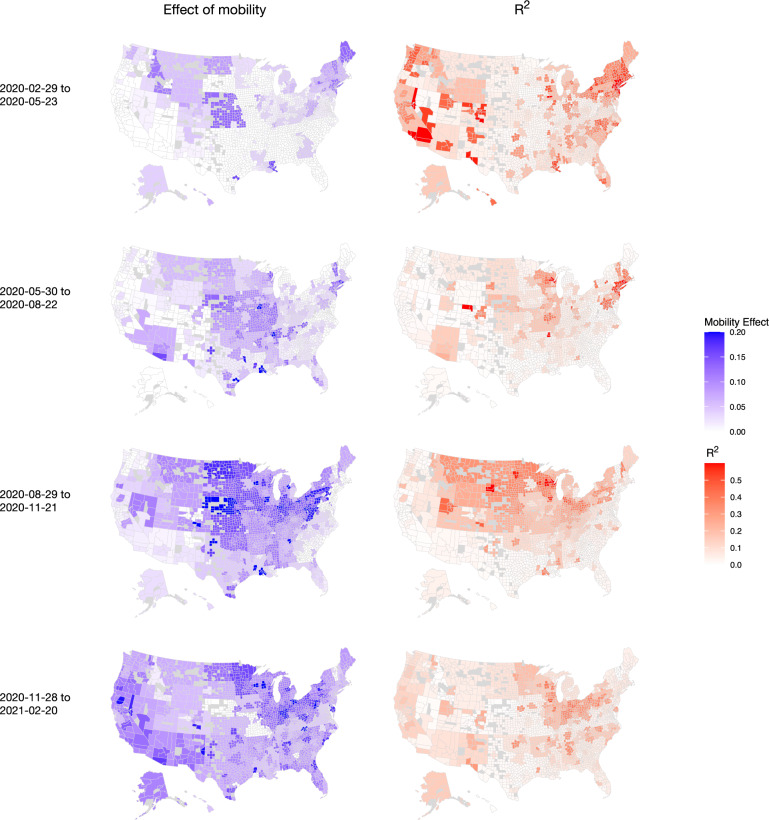
Estimated coefficients and *R*^2^ by CSA across the four waves. Maps created using the R package usmap.

In Fig. 6, we visualize the effect of mobility alongside the corresponding *R*^2^ for each wave and CSA on a map of the US. There is a striking degree of non-stationarity in the estimated effects over time and space. In the first wave, the estimated effect of mobility is close to zero throughout most of the South, as well as much of the West and Midwest. The signal weakens considerably in the second wave, while in the third wave the signal is strongest in the Midwest. Although the estimated effects of mobility sometimes appear strong, as in the fourth wave spanning winter 2020 into early 2021, the corresponding *R*^2^ values are often fairly weak.

### Overly rigid models underfit and wash out spatial and temporal effects

To assess whether our final model can be made simpler without sacrificing accuracy, we consider simpler models that limit mobility’s effect to vary by time and space. We construct an ablation study of six models: letting mobility’s effect vary by CSA, by region, or be fixed nationally; and letting mobility’s effect vary for each wave, or be fixed in time.

**Fig. 7:**
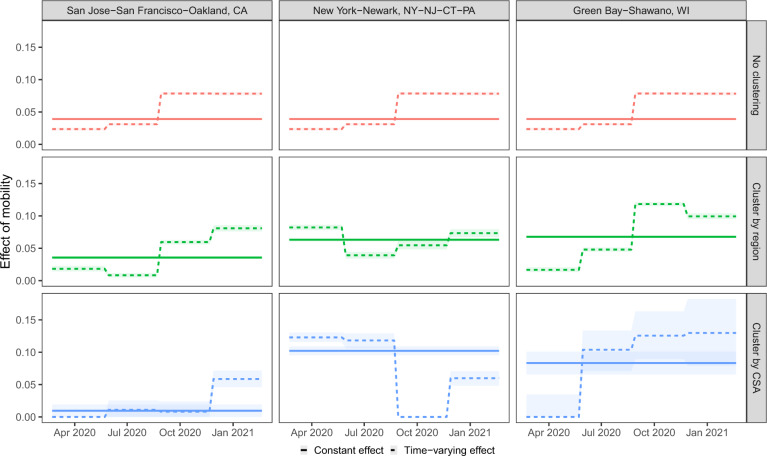
Overly rigid models average over spatiotemporal effects. The estimated effect of mobility for different spatial clustering (rows) and the form of temporal effects (line type) for three illustrative CSAs are displayed. Median (solid lines) and 95% quantiles (shaded) are shown.

For the three example CSAs shown previously, we display the estimated effect of mobility across time for each ablation in Fig. 7. Comparisons of models allowing differential effects of mobility across locations show that rigid national grouping averages over effects visible at finer spatial groupings, such as by region and CSA. Similar limitations are observed with constant temporal effects for mobility. This averaging is not just superficial: our conclusions on the association between mobility and the infection growth rate change. For example, in our final model, we conclude that there is no effect of mobility on the infection growth rate in New York during the third wave. However, all other progressions would conclude that there is a strong association. Likewise, the simpler model that allows mobility’s effect to vary by CSA but forces it to be fixed in time would conclude that New York and Green Bay have very similar associations between mobility and infection rates. However, the final model clearly shows that they are actually quite different, as New York had the strongest association early on while the opposite trend held in Green Bay.

Table 1 tabulates the overall and by region *R*^2^ for each of the six model progressions. As expected, greater flexibility generally results in a higher overall *R*^2^. The greatest differences in *R*^2^ are observed at finer disaggregations: the simplest model has an *R*^2^ of just 19% in the North East, whereas our four-wave CSA model achieves an *R*^2^ of over 40%; indicating that both time-varying coefficients and choice of clustering are critical. In Supplementary Fig. 4 we show that our final model does not overfit.

**Table 1:** *R*^2^ for no spatial clustering, clustering by region, or clustering by CSA, and constant or time-varying mobility coefficients.

Clustering	Mobility effect	Overall	Midwest	Northeast	South	West
None	Constant	0.163	0.201	0.186	0.123	0.175
None	Time varying	0.200	0.245	0.231	0.148	0.233
Region	Constant	0.188	0.275	0.279	0.108	0.158
Region	Time varying	0.216	0.318	0.289	0.125	0.196
CSA	Constant	0.204	0.291	0.303	0.121	0.175
CSA	Time varying	**0.261**	**0.364**	**0.404**	**0.151**	**0.250**

Final model *R*^2^ are shown in bold.

### Assessing the mask effect

On April 4, 2020, the Centers for Disease Control (CDC) began recommending public mask use, a stark reversal of earlier guidance. This led to an increase in mask use across the United States coincident with large drops in mobility. As a result of these concurrent events, mask use and mobility are strongly correlated in the first wave. To facilitate interpretation, we model the association between masks and the infection growth rate as a national effect that is constant across time. All other factors held constant, we estimate an expected 2% decrease in the infection growth rate due to an additive increase in mask adherence of 10%.

To untangle the effect of masks and mobility in the first wave, we compare the *R*^2^ by date in models with and without a mask variable. In the 4-week period following April 4, 2020, we find that overall *R*^2^ increases by approximately 10% when the mask variable is included in the model; see Supplementary Fig. 5 for additional details.

### Conclusions are robust across different mobility data sources

**Fig. 8:**
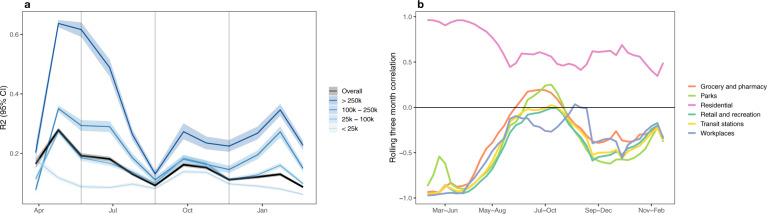
Conclusions are robust across mobility data sources. **a** *R*^2^ obtained using SafeGraph’s completely at home metric in place of the first principle component of Google’s mobility trends dataset. Median (solid lines) and 95% quantiles (shaded) are shown. **b** Rolling median 3-month correlation between SafeGraph’s completely at home mobility metric and each of the six Google mobility indicators. From March–June, absolute correlations were high, indicating consistency between all measures of mobility. However, the strong relationship decayed through May–October suggesting person-to-person contact patterns may not be well captured through coarse cell phone mobility after the initial period.

To assess whether our conclusions are sensitive to the choice of mobility measure, we consider SafeGraph’s completely at home data measure (completely_home_prop_7dav)^10,30^ in place of the first principal component of Google’s mobility indicators. Our conclusions are very similar when using either Google’s or SafeGraph’s mobility measure. Panel (a) of Fig. 8 displays performance, as measured by *R*^2^, over time and by county population. As in Fig. 5, *R*^2^ is highest at the beginning of the pandemic and in high population counties.

In panel (b) of Fig. 8, the rolling 3-month correlation between SafeGraph’s completely at the home measure and each of Google’s six mobility measures is plotted. From March–June 2020, we see correlations with large magnitudes across all variables, providing evidence that stay-at-home orders, lockdown orders, and general uncertainty resulted in a large correlated shift in mobility that is observable across different measures. As a result, during the first wave of the pandemic, any of these mobility measures should have a similar ability to predict infection rates. However, as the pandemic progressed this relationship eroded, potentially suggesting that coarse cell phone measures of mobility began to capture different aspects of mobility and in ways that may not as reliably explain person-to-person contact patterns.

## Discussion

The primary aim of our study is to disentangle how the association between mobility and COVID-19 infection rates varies across time and space. Our work is unique in that it fits time-varying models down to the county level for the vast majority of US counties with an entire year of COVID-19 data. This allows us to much more closely examine when and where broad claims do or do not hold, and to try to assess what drives those patterns.

We find that at an aggregate level, mobility was a strong predictor of COVID-19 weekly infection rates in the first wave, from February 29, 2020 through May 23, 2020. This is similar to findings in other studies, where cell phone mobility was lauded as a strong predictor in the US and globally during the early part of the pandemic^21,22,24,31–33^. A few later studies noted that mobility was markedly less effective as a predictor in the US after the first wave^26,27,34^, which is supported by our findings. We found that the association between mobility and infection rates in the most populous areas largely diminished over the summer and into the fall, then briefly strengthened in late 2020 and into early 2021 before weakening again.

A complete understanding of the relationship between mobility and infection rates remains frustratingly elusive. Importantly, mobility is only a coarse proxy for a desired, but unmeasured quantity: the frequency of risky in-person interactions in a location, which should correlate more directly with infection rates. As in-person interactions changed over the last year due to better mask-wearing, hygiene, and social distancing, mobility data has become confounded and thus a worse proxy for risky interactions. Moreover, the best mobility proxy for risky behavior could differ at different times and locations through the pandemic. We have also demonstrated that as mobility levels have slowly rebounded from extreme decreases seen during the first wave, different mobility measures have become less correlated. This implies that while almost any mobility metric would be a good proxy for risky interactions during extreme mobility decreases, much more care is required to select a proxy as mobility levels veer closer to pre-pandemic levels. We conclude that, while mobility was a reasonable proxy for less-safe practices at first, it was not necessarily stable through time or space.

In terms of modeling, our findings show that models that include mobility need to be either targeted to specific times and places, or include a relationship that varies with time and space. The latter is fundamentally challenging if other complicated relationships, such as with spatiotemporal-varying temperature or mask-usage associations, are included as well. The degrees of freedom quickly overwhelm data that is limited by the collection period and correlations between explanatory variables. All statistical models used to understand relationships between COVID-19 incidence and explanatory variables should be checked for the stability of coefficient values and predictive accuracy across time and space to avoid overfitting and spurious conclusions.

There are several limitations to these conclusions. From a data perspective, we face the fundamental problem of correcting for systematic differences in testing that occur over long periods of time and across locations. Although our use of infection growth rates provides some improvement over directly modeling total inferred infections or unadjusted reported cases, such period-by-period estimates cannot capture longer-term trends in differential testing. While modeling hospitalizations could have addressed such issues, these data are not widely available at the county level. Another limitation arises due to a lack of detailed mask behavior during the initial phases of the pandemic, making the task of disentangling the effect of mobility and masks very difficult; see the Supplementary Materials for an example. Additionally, observed data is often systematically missing and must be imputed, and checking the embedded assumptions in our imputation models is challenging. We also only observe a year of data which makes it impossible to correct for seasonality, such as with the effect of temperature. Finally, we reiterate that different types of mobility were likely better proxies for risky behaviors at differing points during the pandemic. Notably, there has been considerable public discourse about the potential role that schools may have on transmission, but none of the six coarse Google mobility metrics explicitly capture movement to and from schools or universities.

On a modeling side, we choose to pursue statistical models that directly estimate the association between mobility and the infection growth rate. Although these regression models do not allow us to simulate counterfactual scenarios as is possible with compartmental models^21,25,33,35^, such models are restrictive and subject to misspecification. Crucially, infection count data is subject to changing protocols and availability, both of which are confounded by the dynamics of disease spread and can cause identifiability issues for compartmental models. In contrast, our regression framework is relatively easy to calibrate with existing data, and furthermore, the simplicity of these models makes them much more computationally efficient to fit than compartmental models. One other modeling limitation to our work is the simplifying assumption that the effect of mobility can only vary over four 13-week “waves”. While our results did not seem especially sensitive to the exact choice of how waves were defined, this choice may miss subtle effects in certain locations.

In terms of policy, our findings imply that public health officials should not focus exclusively on coarse mobility and must take into account other factors to measure possible transmission events. Conversely, our findings also suggest that there are settings where increased mobility does not necessarily indicate increased rates of transmission. However, the data are far too coarse to indicate what those settings are and what level and type of mobility would be safe. As states loosen mask usage and other restrictions, we might again see a changing effect of mobility. Furthermore, both the proliferation of more transmissible variants of the virus as well as the increasing number of vaccinated people will likely complicate the future relationship between mobility and COVID-19 transmission. These effects were not included in our analyses due to the time periods analyzed, but warrant future investigation.

## Methods

### Overview

Our primary interest is to understand how the relationship between COVID-19 outbreaks and mobility varies across time and geography. Unfortunately, the exact time that new infections occur is never directly observed. Instead, we must rely on noisy observations of the infection incidence such as reported cases, hospitalizations, or deaths. To account for this discrepancy, we estimate the incidence of infections with a newly proposed statistical procedure that robustly estimates the true unknown infection incidence from reported cases^36^. We apply this estimator to daily reported cases for 2951 counties (covering 99.7% of the total population) using data from the New York Times Coronavirus (COVID-19) repository^37^ and the New York City Department of Health COVID-19 repository^38^. We then construct features from aggregated cell phone mobility data, mask-usage surveys, temperature data, and demographic data. These features are used to predict infection growth rates at the county level via a hierarchical Bayesian regression model.

### Infection growth rate as an outcome

We hypothesized that mobility is more likely to correlate with the relative growth of an outbreak rather than with its absolute size. As such, we consider the log growth rate of the estimated incidence curve, henceforth referred to as the growth rate. Specifically, let *r*_*i*,*t*_ be the estimated number of new infections for county *i* that occurred in week *t*. As a unit-less quantity that measures the rate-of-change in the infection rate, the growth rate, *y*_*i*,*t*_, is more robust to differential testing rates than a quantity such as the estimated infection incidence itself. Define the weekly growth rate *y*_*i*,*t*_ as the log ratio of the total infections in the last 2 weeks: ${y}_{i,t}={{\mathrm{log}}}\,\left({r}_{i,t}/{r}_{i,t-1}\right).$

Figure 1 displays the weekly growth rate of infections by geographic divisions, with counties ordered by their CSA. This figure illustrates the heterogeneity in the weekly growth rate: different regions experienced outbreaks of varying severity at different times. Similar temporal trends are observed not only within geographic divisions but also in blocks of counties corresponding to CSAs. Our subsequent modeling choices that involve geographic hierarchies and wave breakpoints are informed by this observed clustering.

### Google mobility data processing

We use Google’s publicly available mobility trends as a surrogate for the frequency of person-to-person contact. Google uses cell phone location data to measure the difference in movement trends during the COVID-19 pandemic from baseline activity before the pandemic for grocery/pharmacy, residential, retail/recreation, workplace, transit, and parks categories. Normal mobility levels are defined for each weekday as the median value of mobility over the 5 week period from January 3, 2020 to February 6, 2020. Normalized daily mobility data for each of the six categories are provided for each county over the course of the pandemic. Additional details about the anonymization procedures, weekend and holiday effects, and general data interpretation are provided by Google at https://support.google.com/covid19-mobility/.

We impute missing temperature and mobility observations from Google’s mobility trends using the Multivariate Imputation by Chained Equations (mice) R package^39^. The MICE algorithm imputes missing values by iteratively fitting a conditional distribution for each variable in a dataset and using it to fill in missing values. This procedure is repeated a number of times until convergence is achieved. We impute values using the predictive mean matching method in mice. We parameterize the conditional distribution for each variable as a linear model, conditioned on the other observed variables. We also allow for a temporal trend per variable (e.g., to allow there to be some trend for mobility) within each US Census division and within each CSA, parameterized by natural cubic splines. This allows each CSA and each division to have its own smoothly varying trend per variable. We fit 25 multiply imputed datasets, and take the mean of these imputations to use for our modeling.

To avoid collinearity in these features, as illustrated in Fig. 3, we fit multilevel regression models with a univariate summary of mobility obtained as the first principal component of Google’s six mobility variables. For interpretation purposes, we enforce a positive first principal loading for workplace mobility, so that higher values of this summary variable indicate more time in public and less time at home.

### Mask featurization

To construct a single measure of mask adherence over the course of the pandemic, we combine survey responses from a few different sources. Pew Research carried out two surveys on June 7, 2020 and August 8, 2020 and released aggregate survey responses at the division level^40^, and the New York Times and Dynata ran county-level surveys from July 2, 2020–July 14, 2020^37^. From September 8, 2020, CMU’s Delphi Epidata group administered and reported state-level daily mask adherence survey responses^30^. We use the COVIDcast Epidata R package to download mask survey responses from CMU’s Delphi Epidata repository.

We define our mask adherence feature piecewise: Between the two Pew survey dates, we linearly interpolate such that the state mask value intersects the average survey response of all counties in a state from the New York Times survey on July 7. The slope of the interpolant is set to the trend between the state’s corresponding June and August Pew division responses. From the value on August 8, we linearly interpolate to the CMU state-level value on September 8. If this results in a decrease in mask adherence between August and September, we instead use a single interpolant from June 7–September 8 defined by two points: the average state-level response from the New York Times survey on July 7, and the state level CMU value on September 8. This monotonicity constraint ensures that the mask adherence level does not increase too quickly between survey dates over the summer.

We further assume zero mask-wearing from the start of the pandemic until one week after the CDC adjusted their mask-wearing recommendation on April 4; prior to this date, the CDC recommended not wearing masks. From April 11 until June 7, the state mask value is equal to the June 7 value.

Supplementary Figure 2 shows the median mask value in each US Census Region and Division; mask compliance roughly increases from April 2020 to February 2021, with high variability across regions and divisions.

### Data aggregation

Mobility, mask use, and temperature time series are constructed by averaging daily measurements within each week. Google’s six mobility metrics and temperature are collected at the county level, and the mask feature is at the state level. The county-level population is an estimate from the 2018 US Census.

### County exclusion criteria

We exclude counties with less than 250 total COVID-19 cases as of the last date considered, February 20, 2021, which removes 176 counties. Next, we exclude counties with extreme growth patterns, where any weekly absolute growth rate exceeds 2 (removing 8 counties), or absolute growth rates exceeds 1.5 and the county has less than 50,000 people (removing 8 counties). These restrictions remove outliers that arise from difficult to model events, such as prison outbreaks in sparsely populated counties.

### Feature selection

In addition to mobility, mask adherence, temperature, and county population, we also considered adjusting for county-level demographic, socioeconomic, and health-related features. However, since these features are constant in time and our model includes a random intercept by CSA, these additional variables only account for intra-CSA variability. Empirically, the inclusion of these variables did not improve performance and made interpretation more difficult. As a result, we excluded these features from our final model.

### Multi-level regression model

We assume that the expected weekly infection growth rate in county *i* at week *t* ∈ {1, …, *N*} is a linear function of population *X*_*i*_, temperature *T*_*i*,*t*_, mask compliance ${C}_{{s}_{i},t}$, and a three week moving average of the first principal component of Google’s six mobility variables (constrained such that workplace mobility’s loading is positive) *M*_*i*,*t*_, at week *t* through a multilevel Bayesian regression model

1 $$\begin{array}{lll}{y}_{i,t}\,=\,{\alpha }_{{c}_{i}}+{X}_{i}\beta +{T}_{i,t}\theta +{C}_{{s}_{i},t}\phi +{M}_{i,t}{\gamma }_{{c}_{i},t}+{\epsilon }_{i,t}\\ \beta ,\theta ,\phi \, \sim \,1\\ \,{\epsilon }_{i,t}\, \sim \,{{{\mathcal{N}}}}(0,{\sigma }_{y}^{2}),\end{array}$$

where *s*_*i*_ is the state of county *i*, the notation “*A* ~ 1” defines an improper flat prior over the reals for the random variable *A*. Log population estimates and weekly temperature observations are each centered by their mean and normalized by twice their sample standard deviation.

To account for geographic clustering observed in the growth rate, we assume that the effect of mobility varies by CSA, i.e., ${M}_{i,t}{\gamma }_{{c}_{i},t}$, where *c*_*i*_ maps county *i* to its CSA (a state pseudo-CSA is created for all counties within a state that do not belong to one of the 175 named CSAs). This allows local information sharing—effectively augmenting missing or incomplete data—between counties within the same CSA. To account for non-stationarity in mobility, we assume that the effect of mobility on the infection rate varies across time. This is encoded through structured time-varying coefficients ${\gamma }_{{c}_{i},t}$. We specify ${\gamma }_{{c}_{i},t}$ through a fixed weight matrix ${{{\bf{W}}}}\in {{\mathbb{R}}}^{N\times R}$ and a cluster-specific vector ${{{{\boldsymbol{\rho }}}}}_{{c}_{i}}$ of dimension *R* ≪ *N* that parameterizes the coefficients

2 $${{{{\boldsymbol{\gamma }}}}}_{{c}_{i},t}={{{{\bf{W}}}}}_{t}{{{{\boldsymbol{\rho }}}}}_{{c}_{i}},$$

where **W**_*t*_ is the *t*th row of the weight matrix **W**. In practice, *N* = 52 as we model a full year of data.

For ease of interpretation, we assume the effect of mobility is piece-wise constant over four waves: February 22, 2020–May 23, 2020; May 30, 2020–August 22, 2020; August 29, 2020–November 21, 2020; November 28, 2020–February 20, 2021. This implies that ${\gamma }_{{c}_{i},t}$ is a piece-wise constant function with three discontinuities and can thus be parameterized by four coefficients that describe the association between mobility and the infection rate in each wave. In the piecewise constant model with *R* = 4 waves, **W** is specified with three fixed knot dates *d*_1_–*d*_3_: the *t*th row of **W** is defined as ${{{{\bf{W}}}}}_{t}=\left[{1}_{(t\le {d}_{1})},{1}_{({d}_{1}\,{ < }\,t\le {d}_{2})},{1}_{({d}_{2}\,{ < }\,t\le {d}_{3})},{1}_{(t \,{ > }\,{d}_{3})}\right]$. Here, 1_*A*_ is an indicator function that equals one if *A* holds, and is zero otherwise. Cluster *c*_*i*_’s **ρ** coefficients are defined as ${{{{\boldsymbol{\rho }}}}}_{{c}_{i}}={\left[{\rho }_{{c}_{i}}^{1},{\rho }_{{c}_{i}}^{2},{\rho }_{{c}_{i}}^{3},{\rho }_{{c}_{i}}^{4}\right]}^{\top }$. In practice, we let *d*_1_ be May 23, 2020, *d*_2_ be August 22, 2020 and *d*_3_ be November 28, 2020, as this evenly splits the 4 waves into groups of 13 weeks each.

We further specify a joint distribution over the coefficients ${\alpha }_{{c}_{i}}$ and ${{{{\boldsymbol{\rho }}}}}_{{c}_{i}}$,

3 $$\left[{\alpha }_{{c}_{i}};{{{{\boldsymbol{\rho }}}}}_{{c}_{i}}^{\top }\right]\mathop{ \sim }\limits^{\,{{\mbox{ind.}}}\,}{{{\mathcal{N}}}}\left(\left[{\alpha }_{0},{{{{\boldsymbol{\rho }}}}}_{0}^{\top }\right],{{{\boldsymbol{\Sigma }}}}\right),$$

where the covariance matrix **Σ** is defined through a scaled correlation matrix **Ω** which is distributed according to the LKJ distribution^41^ with shape parameter equal to two

4 $${{{\boldsymbol{\Sigma }}}}={{{{diag}}}}({{{\boldsymbol{\tau }}}}){{{\boldsymbol{\Omega }}}}{{{{diag}}}}({{{\boldsymbol{\tau }}}}).$$

The scales **τ** are half *t*-distributed with three degrees of freedom. The population-level intercept *α*_0_ is *t*-distributed with three degrees of freedom and **ρ**_0_ ~ 1.

To prevent physically implausible coefficient values, we constrain the coefficients to be positive, that is, ${\gamma }_{{c}_{i},t}\ge 0$ for all *c*_*i*_ and *t*. We enforce this positivity constraint by applying the function *f*(*x*) = max(0, *x*) to all samples from the posterior distribution of ${\gamma }_{{c}_{i},t}$. We found that such post-thresholding generally led to similar estimates when compared to a model where the coefficients ${\gamma }_{{c}_{i},t}$ were log-normally distributed (and thus satisfy ${\gamma }_{{c}_{i},t}\ge 0$), but convergence and sampling time per MCMC iteration was much faster.

### Model training and evaluation

We use the R package brms to obtain posterior samples from our model defined in Eq. (1). Two chains are run for 7000 total iterations; 2000 samples are used for calibration during warm-up. We set the adapt delta and max treedepth settings to 0.9995 and 25, respectively. Every fifth sample is retained for posterior inference. Final models are assessed to ensure convergence: all estimated $\hat{R}$ values are less than 1.05; tail and bulk effective sample sizes are all greater than 1000.

### Reporting summary

Further information on research design is available in the Nature Research Reporting Summary linked to this article.

## Supplementary information

Reporting Summary

Supplementary Information

## Data Availability

We use publicly available data for county-level temperature^42^, Covid-19 case counts^37,38^, mask usage^30,40,43^, Google mobility data^9^, SafeGraph mobility data^10^, and county population^44^. Since the original data used in this work are openly available and the preprocessing scripts are posted at https://github.com/apple/ml-covid-mobility, aggregate data are not available from the corresponding author.

## Code availability

Code to reproduce data pulling/preprocessing, model fitting, and subsequent analyses are available at https://github.com/apple/ml-covid-mobility.
