# Supplementary material for: It’s complicated: characterizing the time-varying relationship between cell phone mobility and COVID-19 spread in the US
Source: NPJ Digit Med. 2021 Oct 27;4:152. doi: 10.1038/s41746-021-00523-3 (PMC8551201; doi:10.1038/s41746-021-00523-3)
Supplement: Supplementary file 1 — Reporting Summary [file 41746_2021_523_MOESM1_ESM.pdf]

nature research

Corresponding author(s): Sean Jewell

Last updated by author(s): September 16, 2021

Reporting Summary

Nature Research wishes to improve the reproducibility of the work that we publish. This form provides structure for consistency and transparency in reporting. For further information on Nature Research policies, see our Editorial Policies and the Editorial Policy Checklist.

Statistics

For all statistical analyses, confirm that the following items are present in the figure legend, table legend, main text, or Methods section.

n/a	Confirmed	
☐	☒	The exact sample size (*n*) for each experimental group/condition, given as a discrete number and unit of measurement
☒	☐	A statement on whether measurements were taken from distinct samples or whether the same sample was measured repeatedly
☒	☐	The statistical test(s) used AND whether they are one- or two-sided<br>*Only common tests should be described solely by name; describe more complex techniques in the Methods section.*
☐	☒	A description of all covariates tested
☒	☐	A description of any assumptions or corrections, such as tests of normality and adjustment for multiple comparisons
☐	☒	A full description of the statistical parameters including central tendency (e.g. means) or other basic estimates (e.g. regression coefficient) AND variation (e.g. standard deviation) or associated estimates of uncertainty (e.g. confidence intervals)
☒	☐	For null hypothesis testing, the test statistic (e.g. *F*, *t*, *r*) with confidence intervals, effect sizes, degrees of freedom and *P* value noted<br>*Give P values as exact values whenever suitable.*
☐	☒	For Bayesian analysis, information on the choice of priors and Markov chain Monte Carlo settings
☒	☐	For hierarchical and complex designs, identification of the appropriate level for tests and full reporting of outcomes
☐	☒	Estimates of effect sizes (e.g. Cohen's *d*, Pearson's *r*), indicating how they were calculated

Our web collection on statistics for biologists contains articles on many of the points above.

Software and code

Policy information about availability of computer code

Data collection	We use publicly available data for county-level temperature [https://goo.gle/covid-19-open-data], Covid-19 case counts [https://github.com/nytimes/covid-19-data; https://github.com/nychealth/coronavirus-data], mask usage [https://github.com/nytimes/covid-19-data/tree/master/mask-use; https://www.pewresearch.org/fact-tank/2020/06/23/most-americans-say-they-regularly-wore-a-mask-in-stores-in-the-past-month-fewer-see-others-doing-it/; https://github.com/cmu-delphi/delphi-epidata], Google mobility data [https://www.google.com/covid19/mobility/], SafeGraph mobility data [https://docs.safegraph.com/docs/social-distancing-metrics; https://github.com/cmu-delphi/delphi-epidata], and county population [https://github.com/JieYingWu/COVID-19USCountylevelSummaries/tree/master/data].
Data analysis	We use R version 4.0.2 along with the following packages.<br>argparse:2.0.3, bayesplot:1.7.2, brms:2.14.0, cmdstanr:0.1.3, covidcast:0.3.0, doParallel:1.0.15, ggdist:2.2.0, gridExtra:2.3, incidental:0.1, lubridate:1.7.9, mice:3.11.0, rstan:2.21.2, readxl:1.3.1:, scales:1.1.1, splines2:0.4.1, tictoc:1.0, tidybayes:2.1.1, tidyverse:1.3.0, usmap:0.5.1.

For manuscripts utilizing custom algorithms or software that are central to the research but not yet described in published literature, software must be made available to editors and reviewers. We strongly encourage code deposition in a community repository (e.g. GitHub). See the Nature Research guidelines for submitting code & software for further information.

Data

Policy information about availability of data

All manuscripts must include a data availability statement. This statement should provide the following information, where applicable:

- Accession codes, unique identifiers, or web links for publicly available datasets
- A list of figures that have associated raw data
- A description of any restrictions on data availability

We use publicly accessible data for county-level temperature [https://goo.gle/covid-19-open-data], Covid-19 case counts [https://github.com/nytimes/covid-19-

data; https://github.com/nychealth/coronavirus-data], mask usage [https://github.com/nytimes/covid-19-data/tree/master/mask-use; https://www.pewresearch.org/fact-tank/2020/06/23/most-americans-say-they-regularly-wore-a-mask-in-stores-in-the-past-month-fewer-see-others-doing-it/; https://github.com/cmu-delphi/delphi-epidata], Google mobility data [https://www.google.com/covid19/mobility/], SafeGraph mobility data [https://docs.safegraph.com/docs/social-distancing-metrics; https://github.com/cmu-delphi/delphi-epidata], and county population [https://github.com/JieYingWu/COVID-19USCountylevelSummaries/tree/master/data].

Field-specific reporting

Please select the one below that is the best fit for your research. If you are not sure, read the appropriate sections before making your selection.

☒ Life sciences ☐ Behavioural & social sciences ☐ Ecological, evolutionary & environmental sciences

For a reference copy of the document with all sections, see nature.com/documents/nr-reporting-summary-flat.pdf

Life sciences study design

All studies must disclose on these points even when the disclosure is negative.

Sample size	No sample size calculation was done since we used observational data in our analyses. We included one year of data, from February 22, 2020 to February 20, 2021, and did not include more recent data while writing the paper.
Data exclusions	From Methods: "We exclude counties with less than 250 total COVID-19 cases as of the last date considered, February 20, 2021, which removes 176 counties. Next, we exclude counties with extreme growth patterns, where any weekly absolute growth rate exceeds 2 (removing 8 counties), or absolute growth rates exceeds 1.5 and the county has less than 50,000 people (removing 8 counties). These restrictions remove outliers that arise from difficult to model events, such as prison outbreaks in sparsely populated counties."
Replication	To verify the reproducibility of our findings, we fit models with many different random initializations across training and test sets. Our findings were stable across these configurations.
Randomization	Randomization is not relevant to our work, as we use retrospective observational epidemiology data sources.
Blinding	Blinding is not relevant to our work, as we use retrospective observational epidemiology data sources.

Reporting for specific materials, systems and methods

We require information from authors about some types of materials, experimental systems and methods used in many studies. Here, indicate whether each material, system or method listed is relevant to your study. If you are not sure if a list item applies to your research, read the appropriate section before selecting a response.

Materials & experimental systems

n/a	Involved in the study	
☒	☐	Antibodies
☒	☐	Eukaryotic cell lines
☒	☐	Palaeontology and archaeology
☒	☐	Animals and other organisms
☒	☐	Human research participants
☒	☐	Clinical data
☒	☐	Dual use research of concern

Methods

n/a	Involved in the study	
☒	☐	ChIP-seq
☒	☐	Flow cytometry
☒	☐	MRI-based neuroimaging
