# Supplementary material for: It’s complicated: characterizing the time-varying relationship between cell phone mobility and COVID-19 spread in the US
Source: NPJ Digit Med. 2021 Oct 27;4:152. doi: 10.1038/s41746-021-00523-3 (PMC8551201; doi:10.1038/s41746-021-00523-3)
Supplement: Supplementary file 2 — Supplementary Information [file 41746_2021_523_MOESM2_ESM.pdf]

Supplementary Materials: It's complicated: characterizing the time-varying relationship between cell phone mobility and COVID-19 spread in the US

Sean Jewell*†, Joseph Futoma†, Lauren Hannah,
Andrew C. Miller, Nicholas J. Foti, Emily B. Fox
Apple, One Apple Park Way, Cupertino, CA 95014

September 23, 2021

Supplementary notes

Data

Estimated infection growth rates by geography and population To illustrate the heterogeneity in the county level estimated log growth rate, in Supplementary Figure 1 we plot the median and empirical 95% quantiles aggregated across county population quantile, US Census Region, and US Census Division.

Although waves of high growth rates followed by low growth rates are evident across aggregations, the log growth rate is a noisy process and thus difficult to generalize trends on the basis of location or population size. There is generally more variability in the log growth rate for smaller population counties.

Estimated coefficients from fitting the model defined in equation (1)

Posterior estimates for population-level parameters are presented in Supplementary Table 1. The mobility wave parameters (i.e. the ρ_0) can be interpreted as the mean effects over all CSAs; these point estimates are comparable to what the effects would be in a model that forces the same association across space and does not allow for differential effects by CSA.

Ablation studies

Results are robust to choice of knot locations. In Supplementary Figure 3, we examine the sensitivity of our results to different knot locations. We consider 125 different models with differing knot dates. In our final model in the main paper, we let d_1 be May 23, 2020, d_2 be August 22, 2020 and d_3 be November 28, 2020, as this evenly splits the 4 waves into groups of 13 weeks each. We considered models where we jittered d_1 by up to 2 weeks

*sean_j@apple.com
†These authors contributed equally

	Estimate	Lower 95% CI	Upper 95% CI	$\hat{R}$	Bulk ESS	Tail ESS
Intercept (α_0)	0.19	0.18	0.20	1.00	1241	1530
Population (β)	0.04	0.03	0.04	1.00	1919	1776
Temperature (θ)	0.03	0.03	0.03	1.00	1882	1604
Mask (ϕ)	-0.19	-0.20	-0.19	1.00	1978	1965
Mobility Wave 1 (ρ_0^1)	0.00	0.00	0.01	1.00	1123	1495
Mobility Wave 2 (ρ_0^2)	0.04	0.03	0.05	1.00	1132	1693
Mobility Wave 3 (ρ_0^3)	0.09	0.07	0.10	1.00	1101	1542
Mobility Wave 4 (ρ_0^4)	0.11	0.10	0.12	1.00	1703	1814
Error scale (σ_y)	0.13	0.13	0.13	1.00	1966	1965

Supplementary Table 1: Posterior population level parameter estimates obtained from 2000 posterior samples (2000 for warm up, and 5000 remaining samples where thinned by saving every 5th sample) in each of two MCMC chains from the model defined in (1).

before or after May 23, 2020 (i.e. we tested $d_1 \in \{2020-05-09, 2020-05-16, 2020-05-23, 2020-05-30, 2020-06-06\}$. Similarly, we jittered d_2 and d_3 by up to 2 weeks before and after their final dates as well, for a total of 125 different knot combinations.

For each model, we compute the overall R^2, R^2 by population, and R^2 by region. As shown in red the R^2 of our final model is roughly centered in each histogram; our final model is not overfit to knot locations.

Averaging over many training and testing splits, our model does not overfit. In order to confirm that our models are not overfit, we ran the final model 100 times each using two different strategies for constructing training and held-out testing sets. First, we created splits by holding out a random 20% of weeks ("random-times"), ignoring geography (i.e. some weeks in a given county will appear randomly in the training set, and some in testing). This specific type of split is less likely to exhibit overfitting, as there will generally be at least some data from every county. Second, we created splits by holding out all data from a random 20% of counties ("random-counties"), fitting the model on the remaining 80%. Supplementary Figure 4 displays the R^2 for each data splitting strategy by week for train and test splits.

When averaging across 100 splits of randomly held-out times, the mean overall R^2 performance is 20.4% (95% CI: (20.2%, 20.6%)) in-sample and 20.3% (95% CI: (19.8%, 20.9%)) out-of-sample. Region-specific R^2 values are: Midwest, 29.1% (28.6%, 29.4%) in-sample, 29.0% (27.9%, 30.1%) out-of-sample; Northeast, 30.1% (29.5%, 30.8%) in-sample, 29.9% (27.7%, 31.9%) out-of-sample; South, 12.2% (12.0%, 12.4%) in-sample, 12.1% (11.5%, 12.6%) out-of-sample; West, 17.7% (17.3%, 18.2%) in-sample, 17.5% (16.5%, 18.6%) out-of-sample.

When averaging across 100 splits of randomly held-out counties, the mean overall R^2 performance is 26.2% (95% CI: (25.7%, 26.9%)) in-sample and 26.7% (95% CI: (24.9%, 29.2%)) out-of-sample. Region-specific R^2 values are: Midwest, 36.6% (35.7%, 37.5%) in-sample, 36.5% (33.2%, 40.9%) out-of-sample; Northeast, 40.7% (38.9%, 42.4%) in-sample, 40.4% (33.9%, 46.8%) out-of-sample; South, 15.2% (14.6%, 16.0%) in-sample, 15.7% (13.4%, 18.1%) out-of-sample; West, 25.4% (24.4%, 26.7%) in-sample, 25.4% (20.0%, 31.4%) out-of-sample.

Adjusting for mask use leads to increased R^2. To assess the effect of mask use, we compare two versions of our final model that differ only in whether or not they include or exclude the mask feature as a global fixed effect. Supplementary Figure 5 shows that the model with masks included leads to substantial increases in R^2 in the first wave (approximately 10%) and moderate increases in the third wave (approximately 4%) over the model with masks excluded. Overall, the R^2 of 27.2% in the final model including masks is about 2% higher than the R^2 of 25.2% in the model excluding masks.

Identification limitations due to mask data: an example

Here we show, through an example, that lack of reliable data limits our ability to assess interactions between mobility patterns and other intervention methods—like mask compliance—on transmission dynamics.

To illustrate this difficultly, consider the estimated effect of mobility in the first wave of the pandemic (Feb 29, 2020—May 23, 2020) in the Los Angeles-Long Beach CSA; shown in the middle panel of Supplementary Figure 6. Although no effect of mobility is estimated in this CSA during the first wave, the estimated fit (right panel) is over 60%. As shown in the left panel, the quality of the fit is due to an increase in mask use during this period. Note that increased mask use coincided with a decrease in mobility over the same period.

In conclusion, even in our relatively simple model class, simultaneous changes in both mask adherence and mobility makes it impossible to disentangle which variable was primarily responsible for the subsequent decrease in infection rates.

Even with more complete data (e.g. perfectly measured mask adherence data each day for this CSA in this time period), identification of these main effects is not guaranteed due to high correlations between increased mask usage and decreased mobility, as well as very little temporal variability in the the dates that mask usage increased. As a result, we are not able to estimate or identify interactions between mobility patterns and intervention methods.

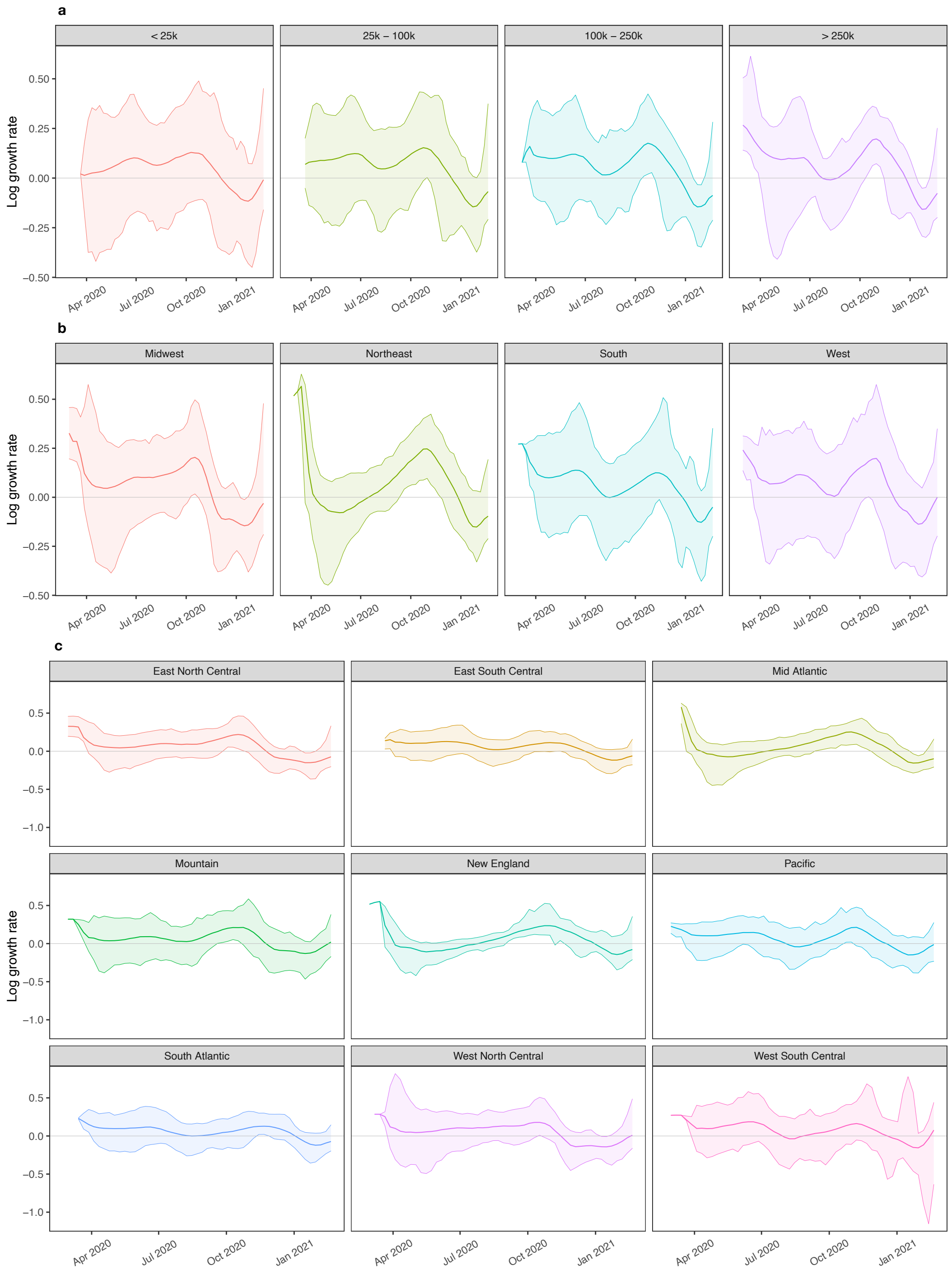

Supplementary Figure 1: **Log growth rate aggregated by population and geography. a.** County population quantile **b.** US Census Region. **c.** US Census Division. Median (solid lines) and 95% quantiles (shaded) are shown.

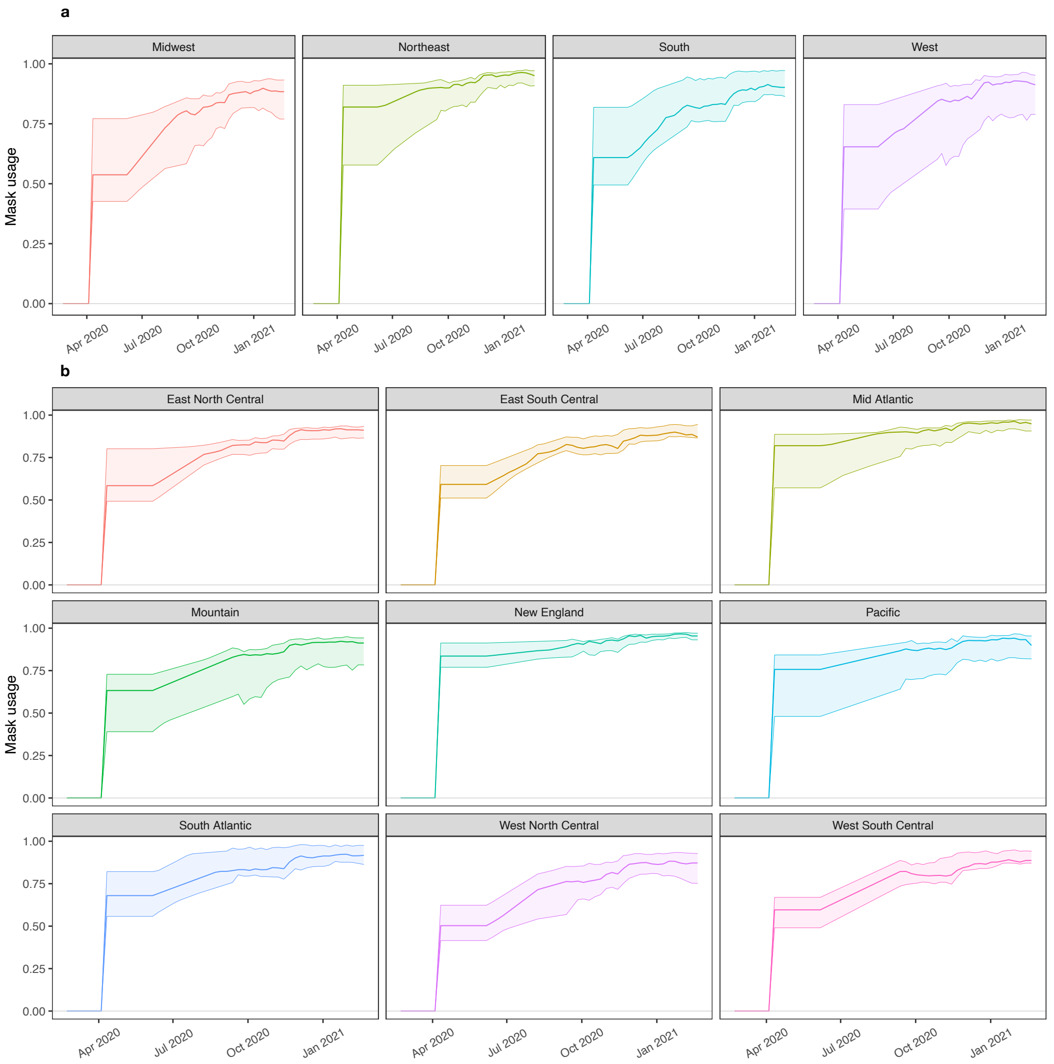

Supplementary Figure 2: **Mask usage by geography. a.** US Census Region. **b.** US Census Division. Median (solid lines) and 95% quantiles (shaded) are shown.

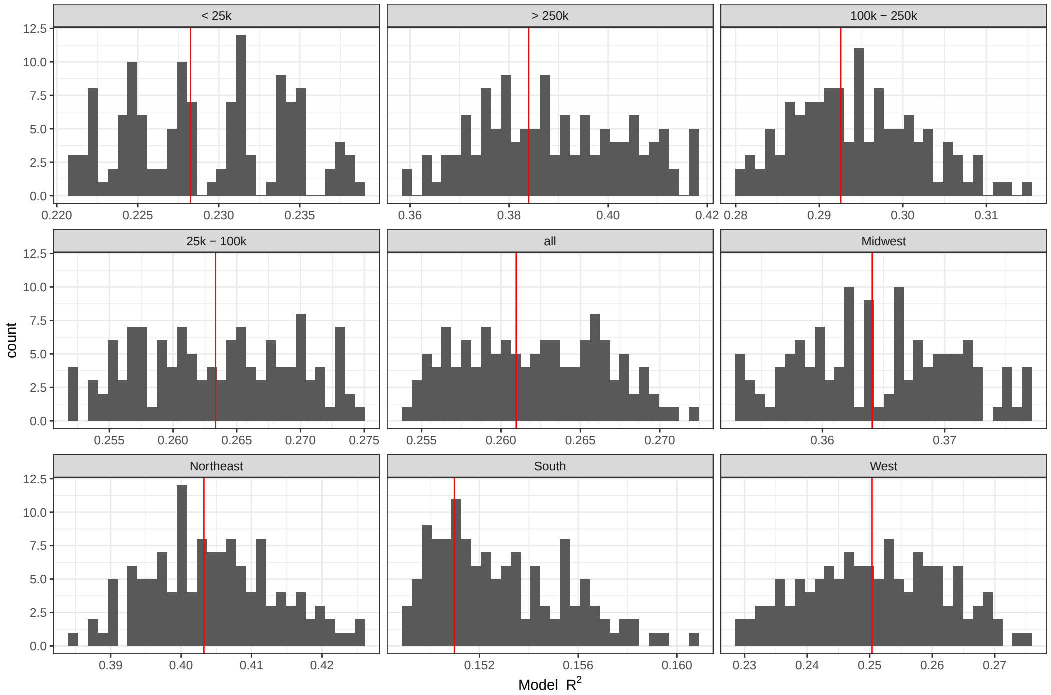

Supplementary Figure 3: **Distribution of R^2 values across 125 different models with slightly different locations for the knots defining the four waves.** Each facet displays a histogram of R^2 across all models on that subset of data (either all of the data, one region, or counties of a certain population size). The red line in each pane shows our final model's R^2. Although performance could be slightly improved, quantitative performance is not very sensitive to the precise choice of knots.

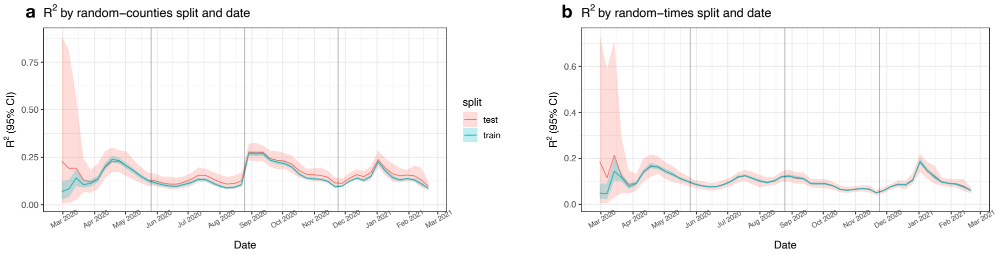

Supplementary Figure 4: **Train/test results, when disaggregating by week. a.** R^2 when 20% of counties are randomly held-out. **b.** R^2 when times are randomly held-out. Median (solid lines) and 95% quantiles (shaded) are shown.

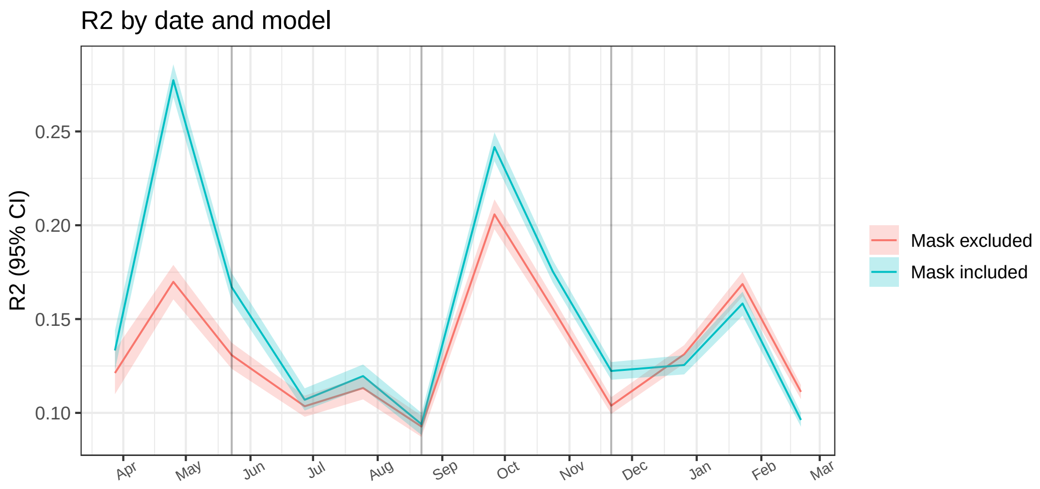

Supplementary Figure 5: R^2 **across time for base model with and without mask variable.** Median (solid lines) and 95% quantiles (shaded) are shown.

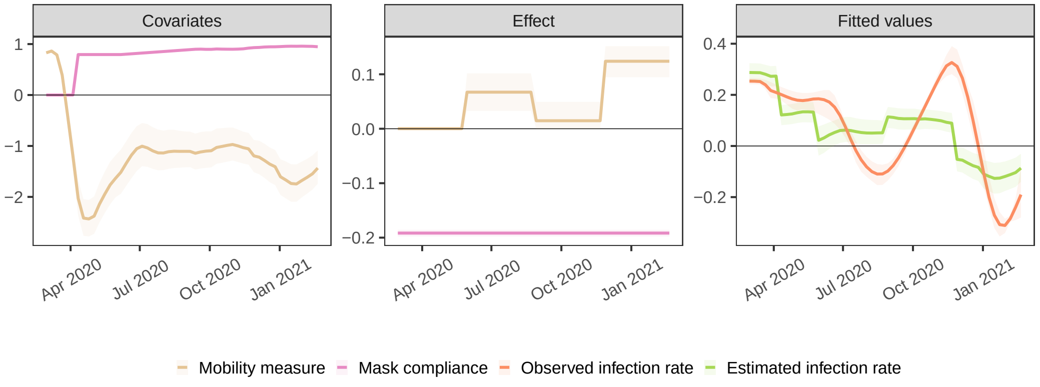

Supplementary Figure 6: **Identification limitations due to mask data.** Mask compliance and mobility covariates (left), estimated effect of masks and mobility (middle), and fitted and observed infection growth rate values (right) for the Los-Angeles-Long Beach CSA. Mobility and mask compliance are highly correlated during the first wave which resulted in no estimated effect of mobility in the first wave. Despite no effect of mobility, the fit during the first wave has an R^2 over 60%.
